# Sensor-Based Structural Health Monitoring of Asphalt Pavements with Semi-Rigid Bases Combining Accelerated Pavement Testing and a Falling Weight Deflectometer Test

**DOI:** 10.3390/s24030994

**Published:** 2024-02-03

**Authors:** Zhen Liu, Bingyan Cui, Qifeng Yang, Xingyu Gu

**Affiliations:** 1 Department of Roadway Engineering, School of Transportation, Southeast University, Nanjing 211189, China; 230208344@seu.edu.cn (Z.L.);; 2 Department of Civil and Environmental Engineering, Rutgers, The State University of New Jersey, Piscataway, NJ 08854, USA; bingyan.cui@rutgers.edu

**Keywords:** sensor monitoring, asphalt pavement, semi-rigid base, accelerated pavement testing, falling weight deflectometer

## Abstract

The Structural Health Monitoring (SHM) of pavement infrastructures holds paramount significance in the assessment and prognostication of the remaining service life of roadways. In response to this imperative, a methodology for surveilling the surface and internal mechanical responses of pavements was devised through the amalgamation of Accelerated Pavement Testing (APT) and Falling Weight Deflectometer (FWD) examinations. An experimental road segment, characterized by a conventional asphalt pavement structure with semi-rigid bases, was meticulously established in Jiangsu, China. Considering nine distinct influencing factors, including loading speed, loading weight, and temperature, innovative buried and layout configurations for Resistive Sensors and Fiber-optic Bragg Grating (FBG) sensors were devised. These configurations facilitated the comprehensive assessment of stress and strain within the road structure across diverse APT conditions. The methodology encompassed the formulation of response baselines, the conversion of electrical signals to stress and strain signals, and the proposition of a signal processing approach involving partial filtering and noise reduction. In experimental findings, the asphalt bottom layer was observed to undergo alternate tensile strains under dynamic loads (the peak strain was ten με). Simultaneously, the horizontal transverse sensor exhibited compressive strains peaking at 66.5 με. The horizontal longitudinal strain within the base and subbase ranged between 3 and 5 με, with the base registering a higher strain value than the subbase. When subjected to FWD, the sensor indicated a diminishing peak pulse signal, with the most pronounced peak response occurring when the load plate was situated atop the sensor. In summary, a comprehensive suite of monitoring schemes for road structures has been formulated, delineating guidelines for the deployment of road sensors and facilitating sustained performance observation over extended durations.

## 1. Introduction

Under the long-term action of natural factors and traffic load [1], the performance of roads continues to decline [2,3,4,5], and the existing road structures have different degrees of damage and functional failure, which will inevitably affect the function of the traffic network [6]. Therefore, it is essential to detect the health status of road structures through time [7]. Road monitoring has traditionally been a manual process that requires a lot of labor and materials [8,9,10]. With the development of electrical and electronic technology, sensor measurement technology is increasingly being applied in engineering measurement [11,12,13]. The stress–strain sensor was initially applied to monitor the performance of bridges [14,15] and dams [16]. Since the Strategic Highway Research Program (SHRP) in the United States in the 1980s [17], a growing number of testers have applied the sensor measurement technology to mechanical response analysis [18,19] or performance monitoring [20,21], and results have been positive.

The earliest use of sensors for road structure physical quantity measurements was in the Minnesota Test Road (MnRoad) and Virginia Test Road in the United States [22]. The Minnesota Test Road embedded a large number of temperature sensors inside road structures to monitor the temperature shrinkage crack problem. Meanwhile, it also included road stress, strain, roadbed humidity, and frost and traffic sensors, which makes it a relatively perfect road test system. In 2006, researchers at Marquette University buried three kinds of strain sensors to study their measurement effectiveness and performance [23], compared their use effects, and recommended a depth and location for the sensor buried. To test the stress and strain response of the flexible pavement for the Center for Pavement and Transportation Technology (CPATT) test road in Canada [24], researchers buried a series of stress, strain, temperature, and humidity sensors in it. According to the monitoring results, the validity of the two-dimensional finite element model was verified.

Currently, there are two mainstream sensors used for road structural monitoring: the resistance sensor [25] and the Fiber-optic Bragg Grating (FBG) sensor [26,27]. Table 1 shows the deficiencies and strengths of two kinds of sensors in road structure monitoring. It can be seen that FBG sensors have advantages in terms of sensitivity and effectiveness, while resistive sensors are more convenient and cheaper. In the process of use, it is necessary to combine the specifics of different sensors to study and formulate the corresponding burying scheme, layout scheme, and signal processing method.

Furthermore, in the use of sensors in road structure monitoring, it is necessary to pay attention to the cooperative deformation of sensors and road materials. A number of scholars have conducted extensive studies on the effectiveness of stress–strain measurements using sensors and the correlation between measured strain and theoretical calculated strain using laboratory tests and numerical simulation. Based on the similarity principle, Tan et al. measured the effectiveness of the FBG sensor and analyzed the strain coordination performance of different FBG sensors in short- and long-term monitoring based on laboratory tests and finite element models [30]. Zhang et al. studied the co-deformation performance of the FBG sensor and asphalt mixture at mesoscale and its influencing factors [31]. Our previous studies, respectively, verified the effectiveness of resistance and FBG sensors in road structure strain monitoring, and proposed their respective signal correction methods, providing ideas for the application of these two types of sensors [32]. These results show that the packaging modulus of existing sensors is generally high, especially in the case of asphalt mixing deformation under the action of high temperatures [33]. Developing a sensor with similar modulus and high sensitivity to the asphalt mixture is still a problem to be solved [34].

In addition, the mechanical response test of the road surface is also an important aspect of road structure monitoring. This method belongs to the non-destructive testing method for pavements. Unlike pavement integrity surveys (internal distress), it calculates the modulus of the road structure on site by measuring the changes in road surface deflection, and then analyzes the mechanical behavior of the road structure according to the monitoring information of the road structure mechanical response, so as to estimate the structural bearing capacity. Among all these kinds of methods, falling weight deflectometer (FWD) is the most commonly used in the deflection testing of road structures [35]. After a comparative analysis, the Federal Highway Administration of the United States believes that FWD is the ideal equipment for pavement bearing capacity assessment, and it is regarded as an important piece of equipment for evaluating pavement bearing capacity in the SHRP program.

Therefore, a comprehensive structural health monitoring initiative was executed for asphalt pavements featuring semi-rigid bases, utilizing a synergistic approach that amalgamated Accelerated Pavement Testing (APT) and FWD tests. This study analyzes the spatial and temporal patterns governing stress and strain within pavement structures by leveraging the measured data obtained from field sensors installed on the test road. It delves into the nuanced variations in pavement response in the face of diverse factors, thereby furnishing substantiating data for developing a mechanical response model for pavement structures. Furthermore, the study employs the FWD test system to scrutinize the temporal and spatial distribution of pulse signals from sensors subjected to the impact load on the road surface. This analysis contributes valuable insights into the dynamic characteristics of the pulse signals, thereby augmenting the understanding of pavement responses under impact loading conditions.

## 2. Objective

The objectives of this study can be summarized as follows:

(1) To analyze the selection principle of road sensors and its influencing factors and to develop a reasonable road structure monitoring scheme;
(2) To explore the processing and correction methods of road sensor data;
(3) To realize a comprehensive analysis of the mechanical responses of road structures.

To fulfill the research objectives, this paper is constructed as follows: Section 3 is the methodology that describes the tested road section, schemes of sensors, and monitoring. Section 4 is the results and discussions, which analyze the results of sensor detection in depth. Finally, Section 5 concludes this study.

## 3. Methodology

### 3.1. Materials and Structures

**Figure 1:**
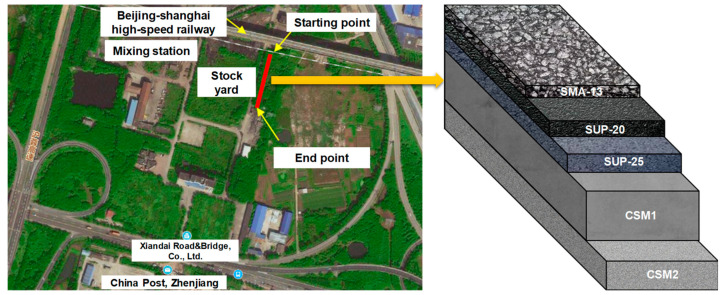
The location, structure, and materials of the APT road test section.

Based on the preliminary investigation and on-site investigation, to ensure the convenience of construction and the scale requirements of the APT site, the project is planned to be near a mixing station and a storage yard with the APT track parallel to the lane next to the storage yard in Zhenjiang, China. The south end of the test road section is connected to the cement hardening storage yard, and the north end is under the Beijing–Shanghai high-speed railway line, as presented in Figure 1.

At present, the asphalt surface thickness of a typical asphalt pavement with semi-rigid bases in Jiangsu Province is 16 to 20 cm. Its specific composition is as follows:

(1) 4 cm stone mastic asphalt (SMA-13) in the upper asphalt layer;
(2) 6 cm superior performing asphalt (SUP-20) in the middle asphalt layer;
(3) 8 cm SUP-25 in the lower asphalt layer.

In addition, cement-stabilized macadam (CSM1) with a cement content of 4.5% was used in the base layer (36 cm), and the CSM2 was used in the subbase layer (20 cm) with 3.0% cement content.

### 3.2. Sensor Monitoring Scheme

#### 3.2.1. Selection for Road Sensors

The selection of road sensors should be considered from the following aspects:

(1) Lifetime: The sensor needs a particular service life to ensure regular operation during observation [36,37].
(2) Size and structures: The size of the sensor needs to be related to the asphalt mixture’s maximum particle size: if the sensor’s size is too small, the deviation of the measurement results may be significant due to the influence of the large particle size aggregate. If the size is too large, it will not only affect the stress state of the material but also lead to more apparent measurement results [38].
(3) Sensitivity and precision: The surface layer’s strain response is generally below 300 με, while the base layer and soil foundation’s stress–strain response is small, requiring high sensitivity and sensor measurement precision [39].
(4) Survival rate during the construction process: Due to the limited effective loading distance of the APT equipment, there is a limit to how many sensors can be buried; thus, sensors with high construction survival rate are preferred [40].
(5) Encapsulation structure and modulus: First, the packaging material has a specific stiffness and strength to ensure that the sensor is not damaged during construction. Then, the packaging material should ensure adequate bonding and deformation co-ordination with the base material. If the packaging material’s modulus is too high, and the sensor’s reinforcement effect is noticeable, this will change the material stress state, resulting in inaccurate measurement results.
(6) Stability and durability: The stability of sensor measurement data, anti-electromagnetic interference ability, and moisture-proof and waterproof ability should be guaranteed. The construction temperature reaches about 150 °C for the asphalt layer sensor, so it need high-temperature resistance [41,42].
(7) Simplicity of construction: This includes the ease of sensor construction and embedding process and the ease of later data wiring and sensor debugging [40].
(8) Coordinated deformation: Due to the modulus variation between sensors and asphalt mixtures, its reinforcement effect cannot be ignored. The co-deformation properties of the two and the effectiveness of stress–strain measurement have been verified in previous studies [32].
(9) Economics: This includes the unit price of the sensor and the price of the corresponding demodulation equipment.

Through the comparison and selection of different manufacturers and models of sensors, and with reference to the sensor selection of Tongji University and Beijing RIOHTrack, the final selected sensor models and manufacturers are shown in Table 2, including traditional resistance sensors and fiber grating sensors.

#### 3.2.2. Arrangement of Road Sensors

**Figure 2:**
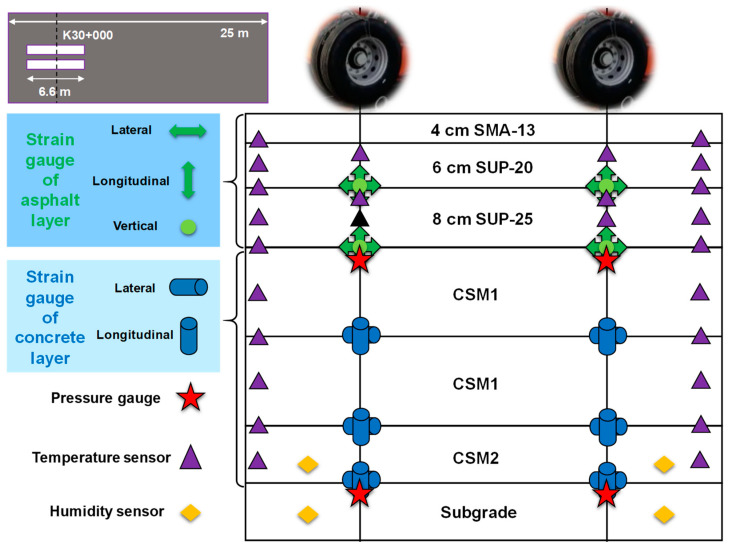
Cross-sectional arrangement of road sensors.

The center line was at pile number K30+000 in the mechanical response test road section. According to the sensor selection scheme in Table 1, the cross-sectional arrangement of sensors within pavement structures is shown in Figure 2. Under the center of the wheel track, lateral, longitudinal, and vertical asphalt strain gauges were buried at the middle and lower asphalt layers’ bottoms. Lateral and longitudinal concrete strain gauges were laid at the upper and lower base layers’ bottoms. The pressure gauges were embedded in the base and the top surface of the subgrade.

**Figure 3:**
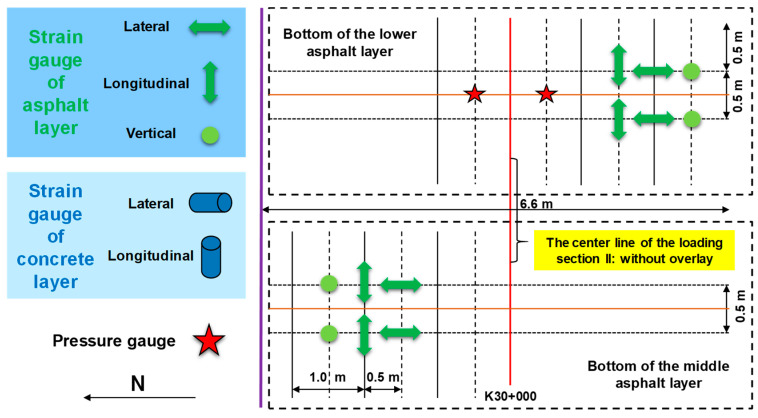
Top view for the arrangement of road sensors at the asphalt layer.

Figure 3 describes the arrangement of road sensors at the asphalt layer from the top view. Specific details referenced our previous works [43].

**Figure 4:**
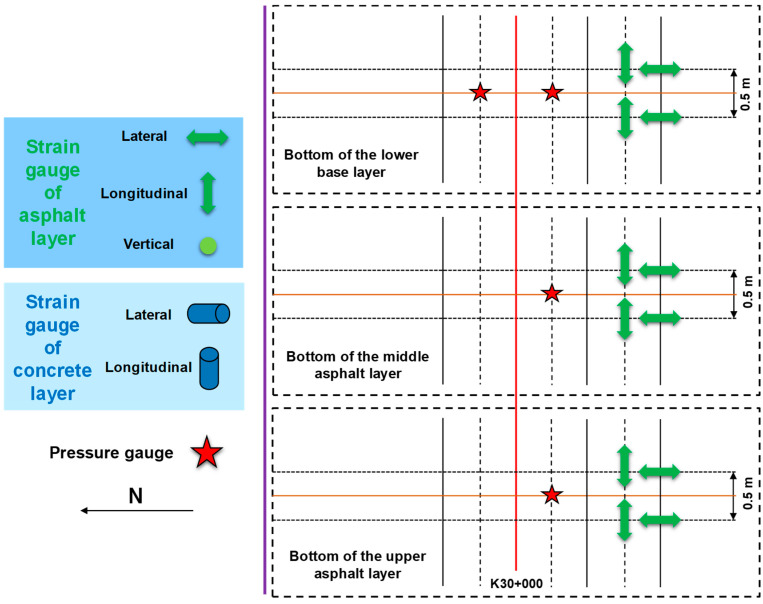
Top view for the arrangement of road sensors at the base layer.

Figure 4 presents the arrangement of road sensors at the base layer from the top view. Four pressure gauges are buried in the bottom of the lower base layer; that is, the top surface of the subgrade base. The strain sensor comprises two lateral and two longitudinal sensors, and a group of strain sensors are buried in the upper and lower base layers and the subbase layer top; the sensor spacing is 50 cm.

#### 3.2.3. Embedding of Road Sensors

The road sensor is a precision measuring instrument. The environment of the sensor buried in the process is relatively harsh, including asphalt concrete and the rolling load of the paving and compacting machinery under high temperatures; this process is also the key to determining whether the sensor is alive. Therefore, the process of embedding the sensor should be combined with the characteristics of the sensor itself and the use of the environment to take appropriate protection measures to ensure the survival of the sensor and adequate bonding with road materials. In this study, the triangulation method was used to locate the buried position of the sensor accurately. The burying process of various sensors is shown in Table 3.

### 3.3. Pavement Testing Scheme

#### 3.3.1. Accelerated Pavement Testing

**Figure 5:**
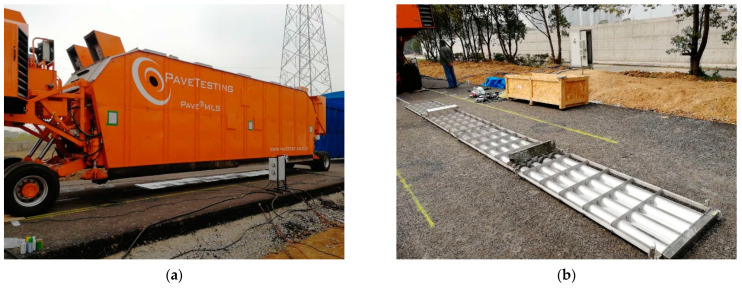
APT device: (**a**) pavement heating system and (**b**) infrared radiation heating system.

As shown in Figure 5, MLS66, the 4th generation linear full-size pavement acceleration loading device with the advantages of self-movement, high-loading frequency, compact structure, and high degree of automation was used for the pavement loading test. The loading parameters of MLS66 were referenced in previous studies [2]: the effective loading length is up to 6 m, the two-wheel axle load is 100 kN, the ground pressure is 0.7 MPa, and the loading speeds are 10 km/h, 15 km/h, and 22 km/h, respectively,

The test equipment allows the road surface to be heated, and the test temperature is controlled by the heating system, with an effective loading length of up to 6 m. The infrared heating system has four heating panels with a total power of 60 kW. The test was loaded 1 million times. After loading, the pavement temperature field was measured by embedding temperature sensors in the high pavement structure layer.

**Figure 6:**
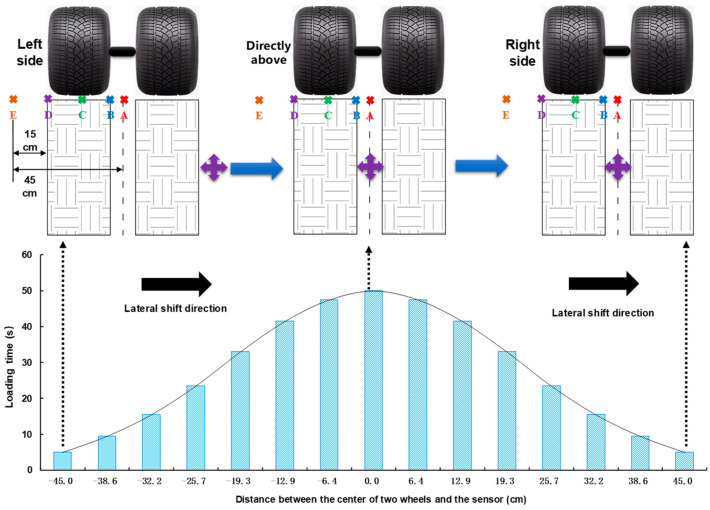
Loading scheme of the lateral movement system of the test road section.

In addition, the lateral movement system equipped with MLS66 can also be used to simulate the wheel track distribution over the road surface cross-section effectively and to investigate the dynamic response of the road surface in time and space. The lateral moving loading scheme is shown in Figure 6. When this system is turned off, the center of the loading twin wheels on one side is directly above the sensor. After the lateral movement system is turned on, the loading wheel moves right and left above the sensor, and the distance is negative when the center of the two wheels is located on the left side of the sensor. The distance is positive when the center of the two wheels is located to the right of the sensor. In vertical cyclic loading, the lateral moving distance of the loading wheel and the loading time is usually distributed, with loading times for loading wheels located above and near the sensor being much longer than loading times for loading wheels located away from the sensor. The output signals of transverse, longitudinal, and vertical strain sensors are collected, and the strain time history curves are drawn during a complete lateral movement period. Five measuring points on the double wheel’s bottom surface were defined to better analyze of the effect of the loading wheel’s position on the strain time curves. The loaded wheel moved laterally and worked in five positions [44].

#### 3.3.2. FWD Test

**Figure 7:**
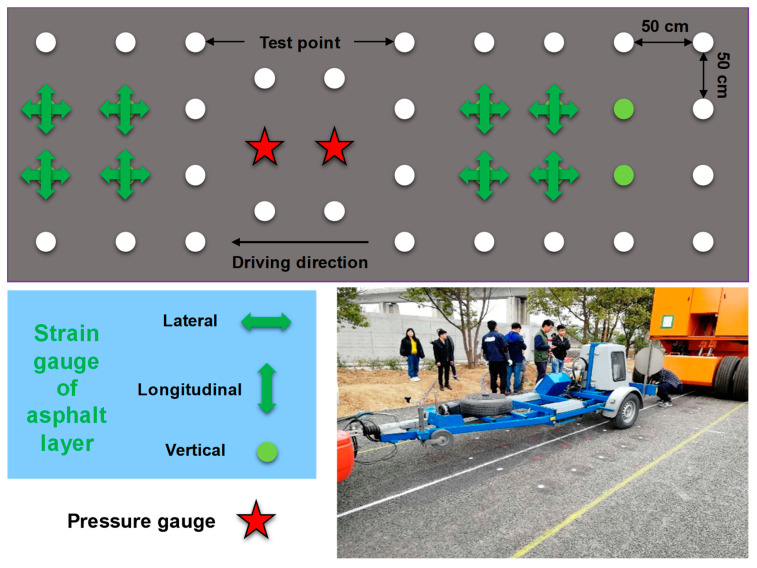
FWD testing process and layout scheme of test points.

We performed the FWD test in the sensor area and the drop weight test 50 cm on each side of the sensor on the test track. As shown in Figure 7, the red measuring point indicates that a strain sensor exists directly below the pavement structure, and the green measuring point indicates that an earth pressure sensor exists directly below the pavement structure. The white measuring point is 50 cm from the outer edge of the sensor. The sensor collects the dynamic response data under the FWD drop hammer in real-time. Dynatest8000 was used as the test device. The radius of the load disk is 300 mm. There are nine displacement sensors with different spacing. The load is divided into two stages: first, the test load is preloaded, and then the formal load is carried out with a 50 kN load.

## 4. Results and Discussions

### 4.1. Sensors Data Acquisition and Processing

**Figure 8:**
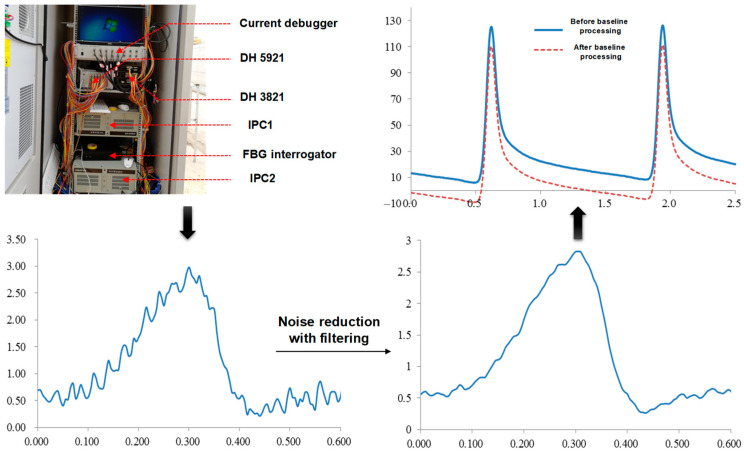
Data acquisition and processing of road sensors.

DH-5921 and DH-3821 devices are used for sensor data processing, and this meets the requirements of high-frequency acquisition of stress and strain at 200 Hz and low-frequency acquisition of temperature and humidity at 2 Hz, respectively. As shown in Figure 8, the sensor readings are interpreted by the system analysis software DHDAS version 2.3 and exported to an EXCEL 2021 (Microsoft) file for subsequent analysis. Because some of the collected data have noise problems, the sensor signal needs to be further filtered and denoised, and the reasonable step value is selected by the moving average method to ensure the authenticity of the data to the greatest extent. It should be noted that for strain sensors, there will be strain accumulation at the initial loading stage, forming a peak phase difference in response. Therefore, it is necessary to subtract the data near the baseline (time t = 0) from this type of sensor’s output signal time history curve to take the data of the modified signal response curve.

Finally, the sensor data in the form of electrical signals should be converted through the conversion coefficient provided by the manufacturer to acquire the response data.

The measurement and conversion methods are similar for the resistance strain sensor, including asphalt concrete horizontal strain gauge, vertical strain gauge, and water stable strain gauge. The strain measurement value of the sensor is the change value of the bridge pressure multiplied by the conversion factor, as shown in Equation (1) [45].

(1) $$\varepsilon_{1}=C_{\varepsilon }\times \frac{V_{i}}{V_{0}}\times 2,$$

where *ε*_1_ represents the measured strain value; *V*_0_ and *V*_i_ represent the measured bridge pressure and volt values, respectively; and *C*_ε_ denotes the conversion coefficient (*C*_ε_ values of the horizontal and vertical sensors are about 0.82 and 0.65, respectively).

The physical quantity measured by the earth pressure sensor is vertical compressive stress, and the sensor manufacturer provides the linear relationship between current and compressive stress, while the signal collected on-site is a voltage signal, which needs to be converted into a current signal and finally converted into a compressive stress signal, as shown in Equation (2) [46].

(2) $$p_{i}=\frac{V_{i}}{\Omega_{0}\times s_{i}},$$

where *p*_i_ represents the measured compressive stress value; Ω_0_ represents the resistance value of the demodulation instrument; and *s*_i_ denotes the sensitivity coefficient sensor (the value of *s*_i_ is generally 0.03202).

The sensitivity of the fiber grating sensor is high, and the sensitivity to temperature cannot be ignored, so the temperature of the sensor must be corrected to obtain the true strain of the sensor. The Fiber Bragg Grating sensor model used was ZX-FBG-S01B, and the signal collected by the fiber demodulation instrument was the central wavelength of the sensor. The strain calculation of the sensor is shown in Equation (3) [47].

(3) $$\varepsilon_{i}=\frac{(\lambda_{i}-\alpha_{T}\cdot \Delta T)-\lambda_{0}}{k}$$

where *ε*_i_ represents the measured strain value; *λ*_i_ and *λ*_0_ represent the measured and initial raster step size, respectively; Δ*T* represents the variation of temperature; *α*_T_ represents the temperature compensation coefficient of sensor, taking 0.2; and *k* denotes the first-term coefficient of strain, taking 0.00093.

### 4.2. Temperature Field Distribution of Asphalt Pavements

**Figure 9:**
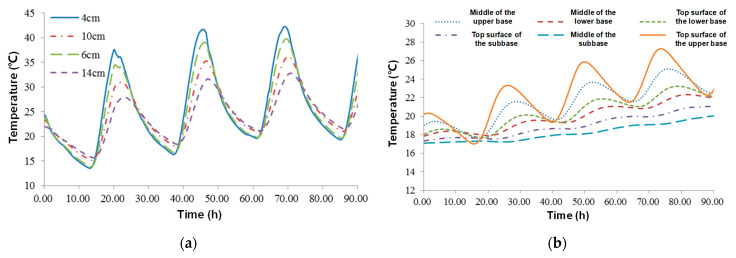
Temperature field distribution of pavement: (**a**) asphalt layer and (**b**) base and subbase layers.

Temperature data have been collected since the end of March 2022. Figure 9a shows the temperature field distribution of the asphalt layer on the test track from 5 p.m. on 4 April to 12 a.m. on 8 April. During the data collection period, the weather was continuously sunny with a maximum temperature of about 30 °C. The overall pavement temperature showed a continuous upward trend. Among all the measuring points, the middle asphalt layer showed the highest temperature, reaching 42.3 °C, which appeared in the afternoon (14:00 to 14:30). In addition, the highest temperature measured by a handheld infrared thermometer reached 60 °C. The middle asphalt layer also showed the lowest temperature (13.5 °C). Thus, the temperature difference in a day was close to 30 °C. With the increase in the depth, the maximum temperature reached by the pavement gradually decreased, and that of the lower asphalt layer still reached 32.8 °C. Moreover, with the increase in the depth, the time of the emergence of the pavement drafting temperature became gradually delayed, and the pavement temperature response had a specific phase difference along the pavement depth. The greater the depth, the greater the phase difference.

Figure 9b describes the change curve of the temperature field of the base and subbase layers with the depth and time of the road surface. The temperature of the base and subbase layers also showed an overall rising trend. The highest temperature also appeared in the top layer, and that of the base layer was 27.3 °C. During the three-day test period, the temperature of the top layer increased by about 5 °C as a whole. With the increase in the structure layer’s depth, the variation range of the temperature in a day gradually decreased. Lower layers of the pavement structure had stable temperatures.

### 4.3. Dynamic Response Analysis of Pavement Structures

#### 4.3.1. Standard Working Conditions

During the experiment, the standard loading conditions were 50 kN of loading weight (two wheels were 100 kN), 20 °C at normal temperature, and 22 km/h maximum loading speed. The dynamic response signals of road sensors under standard loading conditions were collected, and all data were processed according to the standard procedure of sensor signal processing in Section 3.1.

**Figure 10:**
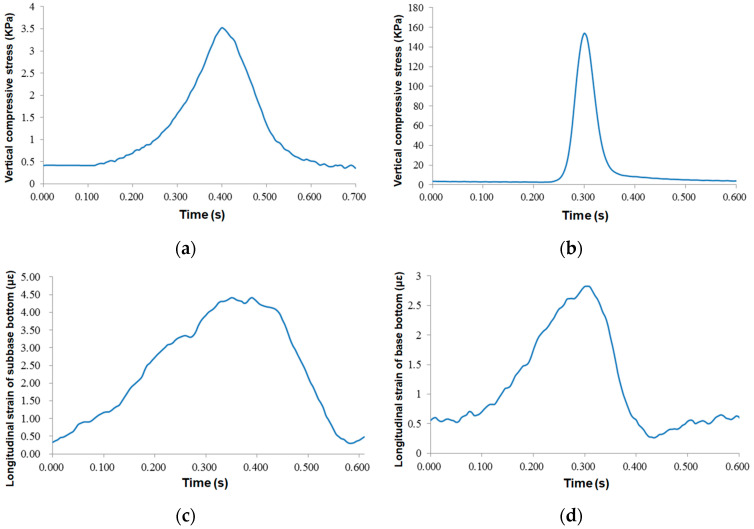
Time-history curves of dynamic responses: (**a**) top surface of subgrade and (**b**) base layer, bottom of (**c**) subbase layer and (**d**) base layer.

Figure 10a,b present the time-history curves of pressure gauges of the subgrade layer. It can be seen that the top surfaces of the base and subgrade layers were under pressure. The response time on the top of the base was short, the peak value changed obviously, and the maximum peak stress was 159 kPa. The maximum compressive stress of the subgrade was 3.55 kPa. Due to the stress diffusion effect, the response time of the subgrade was significantly greater than that of the base—about four times that of the base.

Figure 10c,d show the longitudinal strain response of the subbase and base layers, respectively. As can be seen, both the base and subbase layers were in a state of tensile stress. The maximum longitudinal strain of the base layer was 4.45 με, and the peak strain curve of the base layer was 2.95 με. The response time of the base was twice as long as that of the base strain response. The strain of the subbase layer was greater than that of the base layer, which may be due to the slight difference between the cement content of water-stabilized gravel in the base layer (3.0%) and that of the base layer (4.2%), and the slight difference between the modulus values of the two.

**Figure 11:**
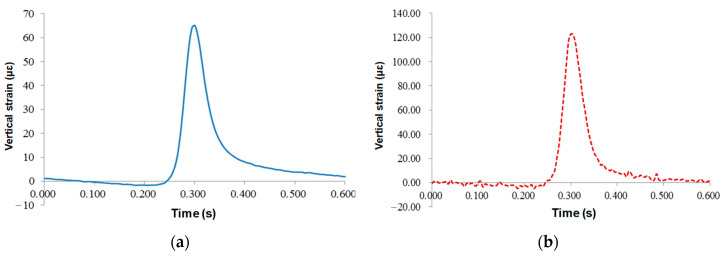
Time-history curves of vertical strains at the lower asphalt layer: (**a**) resistance sensors; (**b**) FBG sensors.

**Figure 12:**
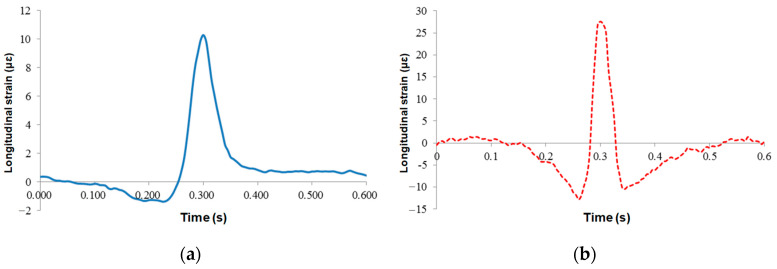
Time-history curves of longitudinal strains at the lower asphalt layer: (**a**) resistance sensors; (**b**) FBG sensors.

Figure 11 and Figure 12 describe the vertical and longitudinal strain responses at the lower asphalt layer. For vertical strains, the response curves of the resistance sensor and FBG sensor were almost synchronized, and the wave peaks were obvious. The maximum vertical strain of the resistance sensor was 66.5 με, and that of the the FBG sensor was 123.2 με, which was twice as large as the resistance strain gauge, indicating that the FBG is highly sensitive.

Before the wheel load arrived, the sensor signal showed a certain concave section, and the asphalt mixture was under pressure; when the wheel load was located on the top surface of the sensors, the asphalt mixture was in tensile stress state. The maximum peak values of the resistive and FBG sensors were 10.28 με and 27.85 με, respectively. When the load left, the sensor still had a short compression time, but the resistance sensor had no prominent concave section, and there was a certain residual strain in the sensor after the vehicle load left. Therefore, the underlying layer of the asphalt mixture was in an alternating state of tension and compression strain under vehicle load. However, the peak value of strain was small, less than the 65 με required for a permanent pavement, indicating that the failure form of this pavement structure is not the fatigue failure of the asphalt bottom.

**Figure 13:**
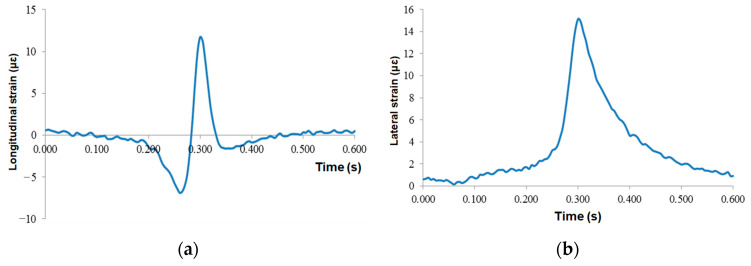
Time-history curves at the bottom of the middle asphalt layer: (**a**) longitudinal strain and (**b**) lateral strain.

The strain responses of the middle asphalt layer are presented in Figure 13. The responses of lateral and longitudinal strain were the same as those of the lower layer, both of which were “compression-tension-compression”, and the maximum tensile strain was 11.7 με, and the concave compression section of the sensor when the vehicle load left was more obvious than that of the lower layer. The lateral strain was always under pressure, and the maximum compressive strain was 15.2 με.

#### 4.3.2. Different Loading Weights

**Figure 14:**
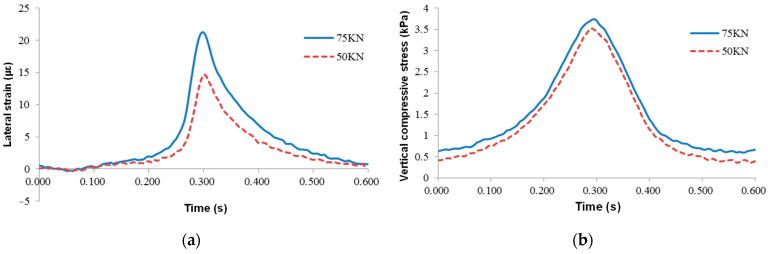
Mechanical response curves under different loading weights: (**a**) lateral strain at the middle asphalt layer’s bottom and (**b**) vertical compressive stress at the top surface of subgrade.

The response of the test road section was measured under standard axle load and 50% overload, respectively. The test conditions were 20 °C temperature and 22 km/h loading speed. As shown in Figure 14, the time-history curve of lateral strain at the middle asphalt layer’s bottom was the same under different coaxial loads, and the sensors were all under pressure. The maximum lateral strain under standard axle load was 14.67 με, and that under overload by 50% was 21.23 με, which increased by 44.7%. It shows that this strain increases almost linearly with the axial load.

The vertical compressive stress on the top surface of the sub-grade was essentially the same under different load weights, showing an overall upward trend. The maximum vertical compressive stress under standard axle load was 3.52 kPa, and that changed to 3.74 kPa after overload by 50%, which only increased by 6.25%.

#### 4.3.3. Different Temperatures

**Figure 15:**
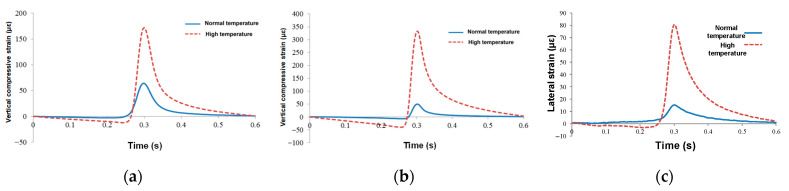
Mechanical response curves under different temperatures: (**a**) vertical compressive strain of the lower asphalt layer, (**b**) vertical compressive strain of the middle asphalt layer, and (**c**) lateral strain of the middle asphalt layer.

Due to the limitation of heating conditions, the maximum temperature of the surface reached 60 °C, and that of the middle layer only reached 40 °C [48]. The average temperature of the lower layer was 35 °C after using the heating system. In the process of high temperature loading, the standard axle load is 50 kN and the maximum loading speed is 22 km/h. As described in Figure 15a, the vertical strain variation trend was the same under different temperatures, and the peak value of the concave section before the vehicle arrives and the peak compressive strain increased greatly. The maximum vertical strain at normal temperature was 64 με, and that at high temperature was 171 με, which increased by 167%. At high temperature, the vertical residual strain increased and the vertical cumulative deformation rate increased obviously. The continuous development of vertical deformation under high temperature is also one of the important reasons for rutting.

In Figure 15b, the vertical strain variation trend of the middle asphalt layer was basically the same as that of the lower asphalt layer at different temperatures, but the average temperature of the middle asphalt layer was higher, and the modulus of the asphalt mixture decreased rapidly and the strain value increased rapidly. The maximum vertical strain at normal temperature was 49.5 με, and that at high temperature was 331.5 με, which increased by more than five times. The middle asphalt layer contributes a lot to the rutting of the road, and the permanent deformation of the middle asphalt layer generally reached 50% of the total rutting of the road structure. The rapid increase in vertical compressive strain and cumulative strain under high temperature conditions is an important part of the rutting of the middle asphalt layer.

Figure 15c presents the middle asphalt layer’s lateral strain under different temperatures. At normal temperature, the middle asphalt layer of the wheel track edge was under pressure under the vehicle load, and the maximum compressive strain was 15.5 με. The strain response curve of the sensor was similar to the vertical strain response of the middle and lower asphalt layers. Before the vehicle load arrived, the middle asphalt layer was subjected to a certain tensile stress. When the load arrived, it was in a state of compression.

#### 4.3.4. Different Loading Speeds

**Figure 16:**
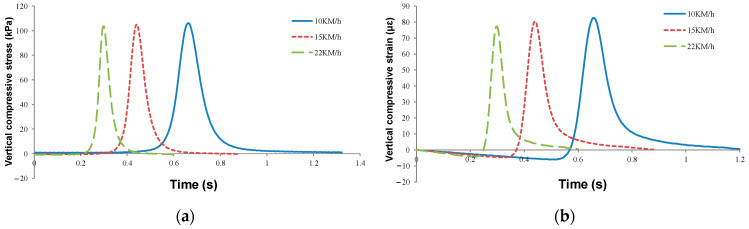
Mechanical response curves under different loading speeds: (**a**) top surface of subgrade and (**b**) lower asphalt layer.

The dynamic response of vehicle load at different driving speeds was simulated. As shown in Figure 16a, the interval time between two adjacent wheels was different at different loading speeds: the interval times between adjacent wheels at 22 km/h, 15 km/h, and 10 km/h were 0.6 s, 0.88 s, and 1.3 s, respectively. Therefore, the action time of vehicle load at any point in the pavement structure increased continuously with decreasing loading speed. However, the vertical compressive stress on the base’s top surface remained unchanged and did not increase with the decrease in loading speed.

As suggested in Figure 16b, the lower layer’s vertical strain increased with decreasing loading speed and with the decrease. The peak vertical strain of the lower layer was 77.2 με under the standard condition of 22 km/h, and that at 15 km/h was 80.3 με, increasing by 4%. The maximum vertical strain at 10 km/h was 82.5 με, 2.7% higher than that at 15 km/h and 6.9% higher at standard conditions. The decrease in loading speed not only increased the action time on the road surface but also increased the road surface strain, and the damage of vehicle load on the road surface increased at a slow speed.

### 4.4. Results Analysis of FWD Tests

#### 4.4.1. Analysis of Sensor Responses

**Figure 17:**
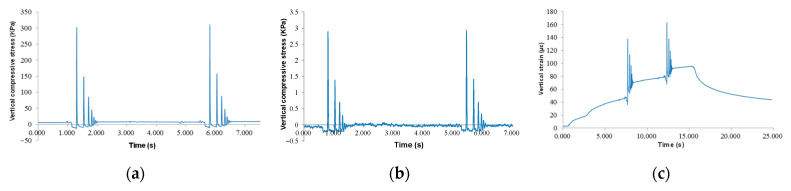
Time-history curves of vertical responses: (**a**) top surface of the base layer, (**b**) top surface of the subgrade, and (**c**) the low asphalt layer.

FWD load plates act on the top surfaces of sensors, causing the greatest sensor response amplitude. Figure 17a shows the response data of pressure gauges. Under two adjacent impact loads, the sensor showed an obvious peak value and decay process. The duration of a single load response was about 0.6 s, which was the same as the load interval time under a loading speed of 22 km/h. After loading, the pressure gauge quickly returned to the initial state, and no obvious residual stress was generated. The time of the two loads was about 4.5 s, and the peak force under the two loads was 310.5 kPa, which was greater than the peak compressive stress generated under the vehicle load. The comparison of each peak value in the decay process shows that the latter peak force was generally half of the previous peak force.

Figure 17b presents the response data of the pressure gauge at the subgrade’s top surface. Compressive stress sensors at the top surface of the subgrade and compressive strain sensors at the top surface of the base were located in the same vertical position and synchronized with each other. The sensor pulse waveform was consistent with that of the compressive stress on the base’s top surface, but the stress response amplitude was small, and the maximum peak stress was only 0.29 kPa, which was smaller than the peak compressive stress under vehicle load. After unloading, the stress of the sensor recovered quickly and no obvious residual stress was generated.

As for the lower asphalt layer in Figure 17c, the obvious pulse peak occurred under both hammer loads, indicating that the lower layer showed vertical compressive strain under FWD load. However, different from pressure gauges, vertical strain sensors had a certain residual strain after each peak strain, and the greater the peak strain, the greater the residual strain. The strain of the vertical sensor tends to increase gradually. The main reason for this phenomenon was that the interval time of the FWD pulse load was short (about 0.2 s), and the strain of the asphalt mixture was not enough to recover to the initial state, resulting in strain accumulation. The maximum peak strain under the first load was 113.2 με, which increased by 66 με compared with the strain before loading. The maximum peak strain under the second load was 163 με, and the strain increased by 83 με. After the FWD device opened the top surface of the sensor, the vertical strain quickly recovered in part, but the residual strain was still 43 με after unloading 10 s.

**Figure 18:**
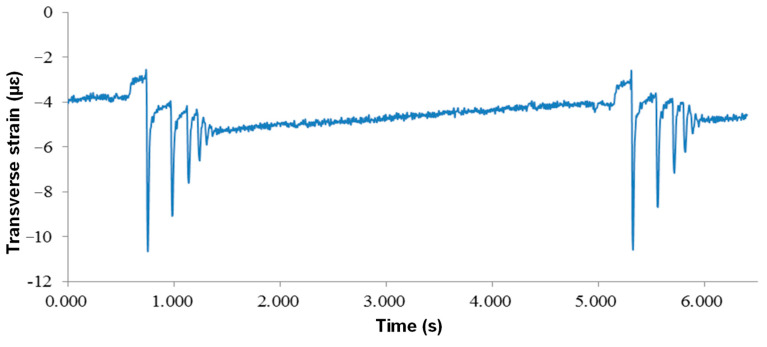
Dynamic response of lateral sensors under FWD partial loads.

In the above sensor responses, the FWD load plate is always located on the top surface. Figure 18 shows the lateral strain sensor’s time history curve when the load plate is 50 cm away from the sensor. The overall performance of the lateral sensor was the compressive strain, and the peak value of compressive strain decreased rapidly under two impact loads. The peak strain of the two loads was 1 to 2 με, and the strain decreased by 2.83 με and 2.66 με, respectively.

#### 4.4.2. Deflection Basin and Back-calculating Modulus

**Figure 19:**
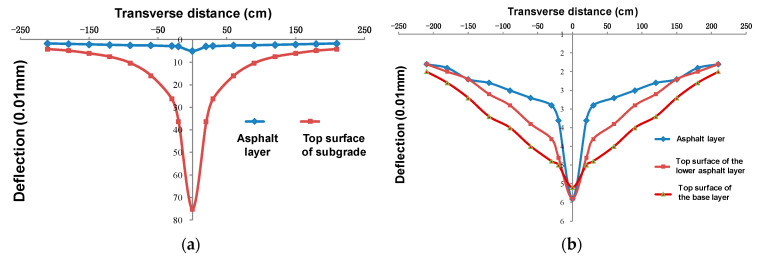
Comparison of deflection basin: (**a**) asphalt layer and subgrade, and (**b**) asphalt layer and base layer.

The deflection basin of the subgrade top surface and the surface layer is shown in Figure 19. The central deflection of the subgrade top surface reached 75.2 (0.01 mm), but it decreased rapidly after the completion of the base laying, reaching about 5.0 (0.01 mm). In addition, there was no obvious rule in the deflection values of the top surface of the base layer, the top surface of the subgrade or the road surface, but the deflection basin curve showed obvious difference: the semi-rigid base’s stiffness was larger, the deflection basin influence range was larger, and it was relatively gentle as a whole. After asphalt layer paving was completed, the slope of deflection basin curve in the range of D_0_ to D_30_ increased significantly, and the displacement mutation phenomenon in D_30_ was more obvious with the asphalt layer’s thickness, while the curved deflection outside the range of D_30_ tended to be flat.

Finally, the back-calculation modulus of pavement material is shown in Table 4. The back-calculation modulus of asphalt concrete was about 4000 to 8000 MPa, and that of the semi-rigid base was about 20,000 to 3000 MPa, but the data were relatively discrete. However, the inverse modulus of the soil foundation was relatively stable, and the inverse modulus was 400 to 500 MPa; it gradually increased with the increasing asphalt layer thickness.

## 5. Conclusions

This study integrated APT and FWD tests to undertake a comprehensive structural health monitoring investigation of an asphalt pavement with semi-rigid bases. The primary conclusions are delineated as follows:

(1) Following the meticulous consideration of nine influencing factors, a road sensor scheme was devised. Additionally, methods were formulated for determining response baselines, converting electrical signals to stress and strain signals, and employing partial filtering and noise reduction in sensor signals. This comprehensive approach not only significantly diminishes signal noise but also preserves the integrity of original data peaks.
(2) The mechanical response of a typical pavement structure and time-history curves under varying loads and environmental conditions were analyzed. Notable changes were observed in the signal period and peak value of the pressure gauge among all sensor signals. The base and subbase strain sensors exhibited some noise signals, while the FBG sensor displayed noise clutter, except for the peak signal.
(3) Under FWD loading, the sensor manifested a diminishing peak pulse signal, with the final peak approximately half of the preceding peak. The sensor’s impulse response was contingent on the position of the sensor relative to the FWD load plate, with the most conspicuous peak response observed when the load plate was positioned on the sensor’s top surface.
(4) After semi-rigid base paving, the pavement structure’s deflection value rapidly decreased. There was a notable difference between the deflection basin curve slope within the D_30_ range and the curve of the base surface and asphalt layer.

During construction, certain sensors experienced a high damage rate. Future efforts should prioritize sensor protection, advocating for fine aggregate protection for sensors. In addition, total station technology can be used to determine the buried position and orientation of sensors.

## Figures and Tables

**Table 1:** Comparison of resistance sensor and FBG sensor in road structural health monitoring [26,28,29].

Type of Road Sensor	Advantages	Disadvantages
Resistance sensor	This sensor has a simple structure and good economy. The resistance strain sensor has the advantages of small size, light weight, high measurement sensitivity, and good frequency characteristics. Able to work in harsh environments of high temperature and pressure.	For large strain, the output signal is weak because of large nonlinearity. Because the output signal is an electrical signal, it is susceptible to interference. The resistance value is affected by changes in temperature.
FBG sensor	Small size and light weight of sensor. Long life, chemical corrosion resistance. Adapts to all kinds of harsh environments. Intrinsically explosion-proof sensor, anti-electromagnetic interference. The number of measuring points is large, can have series-parallel networking. Long-distance transmission, up to 40 km.	The cost of the sensor itself is high. The fiber grating demodulation instrument is expensive, and has certain requirements on the temperature and humidity of the operating environment.

**Table 2:** Selection scheme of road sensors: types, model, and sensitivity.

Types	Models	Sensitivity
Strain gauge of asphalt layer	KM-100HAS	1.0% F.S.
Strain gauge of base layer	KM-100A	1.0% F.S.
Vertical strain gauge	KM-50F	1.0% F.S.
Pressure gauge	OL141D500	0.01% F.S.
Temperature sensor	PT100	±0.3 °C
Humidity sensor	FDS-100	±1%

**Table 3:** Embedding scheme and examples of road sensors.

Types	Embedding Steps	Examples
Strain gauge of asphalt layer	(1) Sensor positioning; (2) Slotted and setting leads; (3) Covering fine aggregates; (4) Compaction molding; (5) Marked and numbered. For the horizontal strain gauges at the asphalt layer, a rubber band should be fixed to the metal rod on both sides of the sensor before embedding to increase the reverse prestress. For the horizontal strain gauges at the base layer, the position and direction of the sensor must be fixed to prevent tilt. For the vertical strain gauges, it is necessary to inject resin at the drilling position or add a bend at the joint and fill it with resin to protect the joint in advance. After solidification, the joint is fixed.	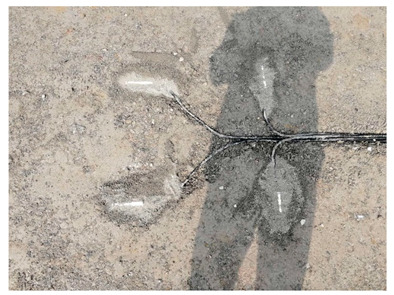 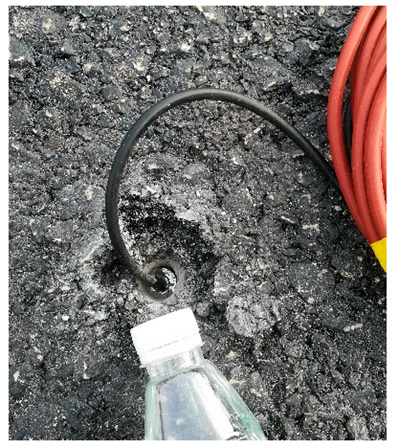
Strain gauge of base layer		
Vertical strain gauge		
Pressure gauge	(1) Determine the center and conductor direction, grooving; (2) Determine the buried position again and draw the boundary of the pressure disc; (3) Covering fine aggregates; (4) Backfilled and compacted.	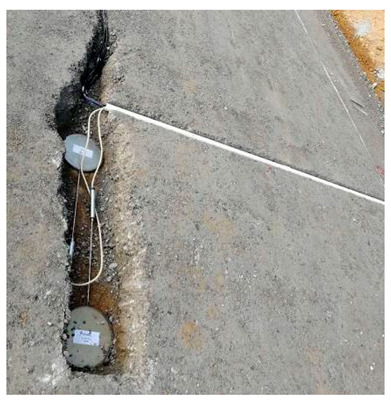
Temperature sensor	(1) Locate the buried sensor; (2) Drill holes along the depth of the road and remove dust and debris; (3) Bundle and secure the temperature sensors; (4) Insert the hole into the desired position; (5) Cement slurry is poured into the hole; (6) The lead leads out of the pavement structure.	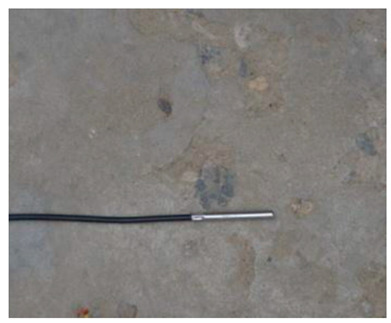
Humidity sensor	(1) Slotted and setting leads; (2) Sensors are implanted in the middle of the subgrade; (3) Covered soil, vibrated, and compacted; (4) Finished the lead and covered the soil again; (5) Static rolling and vibration rolling using roller.	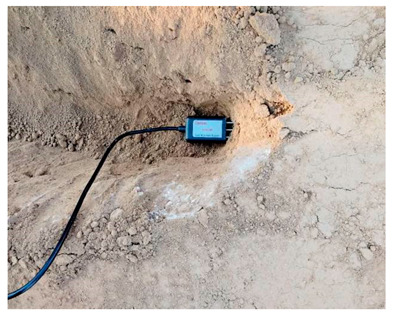

**Table 4:** The back-calculation modulus of the pavement material of the test road (MPa).

FWD Test Point	Statistical Values	Asphalt Layer	Base	Subbase	Subgrade
Top surface of the base layer	Average value	None	23,335	17,012	414
	Mean squared error (MSE)	None	6689	9591	28
Top surface of the lower asphalt layer	Average value	4813	20,545	11,843	460
	MSE	3181	9936	5524	24
Top surface of the middle asphalt layer	Average value	7136	24,881	24,718	476
	MSE	1699	9248	5199	23
Road surface	Average value	7587	31,954	17,582	502
	MSE	1437	9787	7625	33

## Data Availability

Restrictions apply to the availability of these data. Data can be obtained from the corresponding author, on request.

## References

[1] de Silva A., Ranasinghe R., Sounthararajah A., Haghighi H., Kodikara J. (2024). Beyond Conventional Monitoring: A Semantic Segmentation Approach to Quantifying Traffic-Induced Dust on Unsealed Roads. Sensors.

[2] Liu Z., Gu X., Ren H., Li S., Dong Q. (2023). Permanent Deformation Monitoring and Remaining Life Prediction of Asphalt Pavement Combining Full-Scale Accelerated Pavement Testing and FEM. Struct. Control Health Monit..

[3] Chen F., Balieu R., Kringos N. (2016). Potential influences on long-term service performance of road infrastructure by automated vehicles. Transp. Res. Rec..

[4] Wei Z., Jia Y., Wang S., Zhou Z., Zhang Z., Wang X., Huang X., Gao Y. (2022). Influence of iron tailing filler on rheological behavior of asphalt mastic. Constr. Build. Mater..

[5] Jia M., Xu J., Gao C., Mu M., E G. (2023). Long-Term Cross-Slope Variation in Highways Built on Soft Soil under Coupling Action of Traffic Load and Consolidation. Sustainability.

[6] Ho H., Nishio M. (2022). Evaluation of dynamic impact factor of existing bridges with road surface damages based on dynamic response under traffic flow loading. Struct. Infrastruct. Eng..

[7] Mishra R., Gupta H.P., Dutta T. (2020). A road health monitoring system using sensors in optimal deep neural network. IEEE Sens. J..

[8] Dong D., Li Z. (2021). Smartphone Sensing of Road Surface Condition and Defect Detection. Sensors.

[9] Liu Z., Gu X., Dong Q. (2023). Permanent Deformation Evaluation and Instability Prediction of Semi-rigid Pavement Structure Using Accelerated Pavement Testing and Finite Element Method. J. Test. Eval..

[10] Al-Ali A.R., Beheiry S., Alnabulsi A., Obaid S., Mansoor N., Odeh N., Mostafa A. (2024). An IoT-Based Road Bridge Health Monitoring and Warning System. Sensors.

[11] Wang M.L., Lynch J.P., Sohn H. (2014). Sensor Technologies for Civil Infrastructures.

[12] Das S., Saha P. (2018). A review of some advanced sensors used for health diagnosis of civil engineering structures. Measurement.

[13] DiLorenzo T., Yu X. (2023). Use of ice detection sensors for improving winter road safety. Accid. Anal. Prev..

[14] Wang D., Zhang J., Zhu H. (2015). Embedded electromechanical impedance and strain sensors for health monitoring of a concrete bridge. Shock Vib..

[15] Liang X. (2024). Enhancing Seismic Damage Detection and Assessment in Highway Bridge Systems: A Pattern Recognition Approach with Bayesian Optimization. Sensors.

[16] Scaioni M., Marsella M., Crosetto M., Tornatore V., Wang J. (2018). Geodetic and remote-sensing sensors for dam deformation monitoring. Sensors.

[17] Bahia H.U., Anderson D.A. (1995). Strategic highway research program binder rheological parameters: Background and comparison with conventional properties. Transp. Res. Rec..

[18] Gong M., Zhou B., Chen J., Sun Y. (2021). Mechanical response analysis of asphalt pavement on concrete curved slope bridge deck based on complex mechanical system and temperature field. Constr. Build. Mater..

[19] Wang T., Dong Z., Xu K., Ullah S., Wang D., Li Y. (2022). Numerical simulation of mechanical response analysis of asphalt pavement under dynamic loads with non-uniform tire-pavement contact stresses. Constr. Build. Mater..

[20] Barriera M., Pouget S., Lebental B., Van Rompu J. (2020). In situ pavement monitoring: A review. Infrastructures.

[21] Dawson T., Baladi G., Musunuru G., Prohaska M., Jiang Y. (2016). Global procedure for temperature adjustment of measured pavement deflection data: Based on the long-term pavement performance Seasonal Monitoring Program. Transp. Res. Rec..

[22] Worel B., Vrtis M., Buzz Powell R. (2020). Guidance for the Next Generation Accelerated Pavement Testing Facilities. Accelerated Pavement Testing to Transport Infrastructure Innovation.

[23] Crovetti J.A., Hornyak N.J., Schabelski J.P., Newman D.E. (2007). Marquette Interchange Perpetual Pavement Instrumentation Project: Phase I Final Report.

[24] Tighe S., Falls L.C., Doré G. (2007). Pavement performance evaluation of three Canadian low-volume test roads. Transp. Res. Rec..

[25] Rasol M., Schmidt F., Ientile S., Adelaide L., Nedjar B., Kane M., Chevalier C. (2021). Progress and monitoring opportunities of skid resistance in road transport: A critical review and road sensors. Remote Sens..

[26] Braunfelds J., Senkans U., Skels P., Janeliukstis R., Porins J., Spolitis S., Bobrovs V. (2022). Road Pavement Structural Health Monitoring by Embedded Fiber-Bragg-Grating-Based Optical Sensors. Sensors.

[27] Kashaganova G., Kozbakova A., Kartbayev T., Balbayev G., Togzhanova K., Alimseitova Z., Orazaliyeva S. (2023). Research of a Fiber Sensor Based on Fiber Bragg Grating for Road Surface Monitoring. Electronics.

[28] Braunfelds J., Senkans U., Skels P., Janeliukstis R., Salgals T., Redka D., Lyashuk I., Porins J., Spolitis S., Haritonovs V. (2021). FBG-based sensing for structural health monitoring of road infrastructure. J. Sens..

[29] Wang J., Han Y., Cao Z., Xu X., Zhang J., Xiao F. (2023). Applications of optical fiber sensor in pavement Engineering: A review. Constr. Build. Mater..

[30] Tan Y., Wang H., Ma S., Dong Z., Shao X., Chen F. (2013). Calibration Method of FBG Temperature Sensor Used in Asphalt Pavement. J. Build. Mater..

[31] Zhang X.-F., Qian Z.-D., Zhang M., Chen L.-L. (2019). Numerical Simulation for Synergetic Deformation of Optical Fiber Sensor and Asphalt Mixture. Ksce J. Civ. Eng..

[32] Liu Z., Gu X.Y., Wu C.Y., Ren H., Zhou Z., Tang S. (2022). Studies on the validity of strain sensors for pavement monitoring: A case study for a fiber Bragg grating sensor and resistive sensor. Constr. Build. Mater..

[33] Ye Z., Cai Y., Liu C., Lu K., Ildefonzo D.G., Wang L. (2022). Optimization of Embedded Sensor Packaging Used in Rollpave Pavement Based on Test and Simulation. Materials.

[34] Wei Z., Jia Y., Wang S., Li Z., Li Y., Wang X., Gao Y. (2022). Utilization of iron ore tailing as an alternative mineral filler in asphalt mastic: High-temperature performance and environmental aspects. J. Clean. Prod..

[35] Liu Z., Yang Q., Gu X. (2023). Assessment of Pavement Structural Conditions and Remaining Life Combining Accelerated Pavement Testing and Ground-Penetrating Radar. Remote Sensing.

[36] Wang D., Yi Z., Ma G., Dai B., Yang J., Zhang J., Yu Y., Liu C., Wu X., Bian Q. (2022). Two-channel photonic crystal fiber based on surface plasmon resonance for magnetic field and temperature dual-parameter sensing. Phys. Chem. Chem. Phys..

[37] Ruseruka C., Mwakalonge J., Comert G., Siuhi S., Perkins J. (2023). Road Condition Monitoring Using Vehicle Built-in Cameras and GPS Sensors: A Deep Learning Approach. Vehicles.

[38] Tan X., Abu-Obeidah A., Bao Y., Nassif H., Nasreddine W. (2021). Measurement and visualization of strains and cracks in CFRP post-tensioned fiber reinforced concrete beams using distributed fiber optic sensors. Autom. Constr..

[39] Tan X., Bao Y. (2021). Measuring crack width using a distributed fiber optic sensor based on optical frequency domain reflectometry. Measurement.

[40] Zhu W., Yi Y., Yi Z., Bian L., Yang H., Zhang J., Yu Y., Liu C., Li G., Wu X. (2023). High confidence plasmonic sensor based on photonic crystal fibers with a U-shaped detection channel. Phys. Chem. Chem. Phys..

[41] Tan X., Bao Y., Zhang Q., Nassif H., Chen G. (2021). Strain transfer effect in distributed fiber optic sensors under an arbitrary field. Autom. Constr..

[42] Wang D., Zhu W., Yi Z., Ma G., Gao X., Dai B., Yu Y., Zhou G., Wu P., Liu C. (2022). Highly sensitive sensing of a magnetic field and temperature based on two open ring channels SPR-PCF. Opt. Express.

[43] Liu Z., Gu X.Y., Ren H., Zhou Z., Wang X., Tang S. (2022). Analysis of the dynamic responses of asphalt pavement based on full-scale accelerated testing and finite element simulation. Constr. Build. Mater..

[44] Ling J., Wei F., Zhao H., Tian Y., Han B., Chen Z.A. (2019). Analysis of airfield composite pavement responses using full-scale accelerated pavement testing and finite element method. Constr. Build. Mater..

[45] Zhang Q., Li R., Yuan H. (2024). Novel measurement method for full-field bridge strain and displacement with limited long-gauge strain sensors. Meas. Sci. Technol..

[46] Han J.-H., Min S.J., Lee E.-S., Kim J.H., Min N.K. (2023). Half-Bridge Silicon Strain Gauges with Arc-Shaped Piezoresistors. Sensors.

[47] Tao J., Chen Y., Lu J. (2018). Method of the Cavity Length Demodulation for Optical Fiber F-P Sensors Based on Sparse Fast Fourier Transform. Chin. J. Lasers.

[48] Liu Z., Zhou Z., Gu X., Sun L., Wang C. (2023). Laboratory evaluation of the performance of reclaimed asphalt mixed with composite crumb rubber-modified asphalt: Reconciling relatively high content of RAP and virgin asphalt. Int. J. Pavement Eng..

